# Method and reliability of measuring midurethral area and echogenicity, and changes during and after pregnancy

**DOI:** 10.1007/s00192-018-3580-z

**Published:** 2018-03-12

**Authors:** Maria K. van de Waarsenburg, Nienke E. van Hoogenhuijze, Anique T. M. Grob, Karlijn J. Schweitzer, Mariëlla I. J. Withagen, Carl H. van der Vaart

**Affiliations:** 1 Department of Obstetrics and Gynecology, University Medical Centre Utrecht, Heidelberglaan 100 Huispostnummer F05.126, 3584 CX Utrecht, The Netherlands; 2 MIRA Institute for Biomedical Technology and Technical Medicine, University of Twente, Enschede, The Netherlands

**Keywords:** Transperineal ultrasound, 3D/4D, Urethra, Area, Echogenicity

## Abstract

**Introduction and hypothesis:**

Internal closure of the urethral sphincter is one of the mechanisms in maintaining continence. Little is known about changes in the urethral sphincter during pregnancy. We designed this study to develop a reliable method to measure the area and mean echogenicity of the midurethra during and after pregnancy and to assess changes over time.

**Methods:**

Two observers independently segmented the urethra as follows: in the sagittal plane, the urethra was positioned vertically, the marker was placed in the middle section of the lumen of the urethra, and eight tomographic US images of 2.5 -mm slices were obtained. The central image was selected, and area and mean echogenicity were calculated automatically. Intra- and interobserver reliability were determined by intraclass correlation coefficients (ICC) and their 95% confidence intervals (CI). Two hundred and eighty women underwent TPUS at 12 weeks and 36 weeks of gestation and 6 months postpartum, and 3D/4D transperineal ultrasound (TPUS) images of 40 pregnant nulliparous women were used for the reliability study. Paired* t* tests were used to assess changes in echogenicity and area.

**Results:**

The ICC for measuring the area was substantial, at 0.77 and for measuring mean echogenicity was almost perfect, at 0.86. In the total study group (*n* = 280), midurethral area and mean echogenicity were significantly lower 6 months after delivery compared with 12 and 36 weeks of gestation.

**Conclusions:**

Our protocol for measuring area and mean echogenicity of the midurethra is reliable. This study indicates that structural changes in the midurethraoccur during pregnancy.

## Introduction

Transperineal ultrasound (TPUS) can be used to assess urethral support by measuring urethral and bladder-neck (BN) mobility during and after pregnancy [1–8]. TPUS is superior in imaging the pelvic floor, as all anatomical structures are well visualized. It is also more patient friendly than transvaginal (TVUS) and transurethral (TUS) US, especially in pregnant women. In contrast to studies on BN and urethral mobility, little is known about possible changes in urethral sphincter muscle during pregnancy. Urethral sphincter function depends on muscle quantity (volume/thickness), [9, 10] composition [proportion of muscle fibers and extracellular matrix (ECM)], and innervation [11], and smaller volume/thickness of the urethral sphincter, as measured by TVUS, correlates with SUI [12, 13].

Structural composition of muscle tissue can be assessed indirectly by measuring echogenicity (greyscale with a range 0–255). [14, 15] Echogenicity reflects the ratio between muscle cells (dark on ultrasound) and ECM. In general, a low echogenicity reflects a predominance of muscle fibers, and a high echogenicity reflects more ECM. [14] Mitterberger et al. investigated the urethra with using TUS [16]. Lesions, presenting as hyperechoic structures were seen more frequently in the urethral sphincter of incontinent versus continent women [16].

We designed this study to develop a reliable method to measure the and mean echogenicity of the midurethra as a representation of the urethral sphincter during and after pregnancy using 3D/4D TPUS and to assess changes over time.

## Methods

This study was a subanalysis of a prospective observational study on the association between urogenital symptoms and pelvic floor anatomy during and after pregnancy. [3] Two hundred and eighty nulliparous women with a singleton pregnancy and good knowledge of the Dutch language were recruited. All participants underwent 3D/4D TPUS assessment with an empty bladder at 12 and 36 weeks’ gestation and 6 months after delivery. Volume-imaging data sets were obtained at rest, on maximum pelvic floor muscle contraction, and on maximum Valsalva maneuver. Exclusion criteria were a medical history of urinary or fecal incontinence, anti-incontinence or prolapse surgery, neurological disorders/connective tissue diseases, and inability to perform maximal Valsalva maneuver due to pulmonary or heart disease. This study was approved by the institutional Human Research Ethics Committee (reference 08–299); all women gave written informed consent. For the reliability study, a random selection of US images at 12 weeks’ gestation in 40 participants with an uncomplicated pregnancy and vaginal delivery was used.

**Fig. 1:**
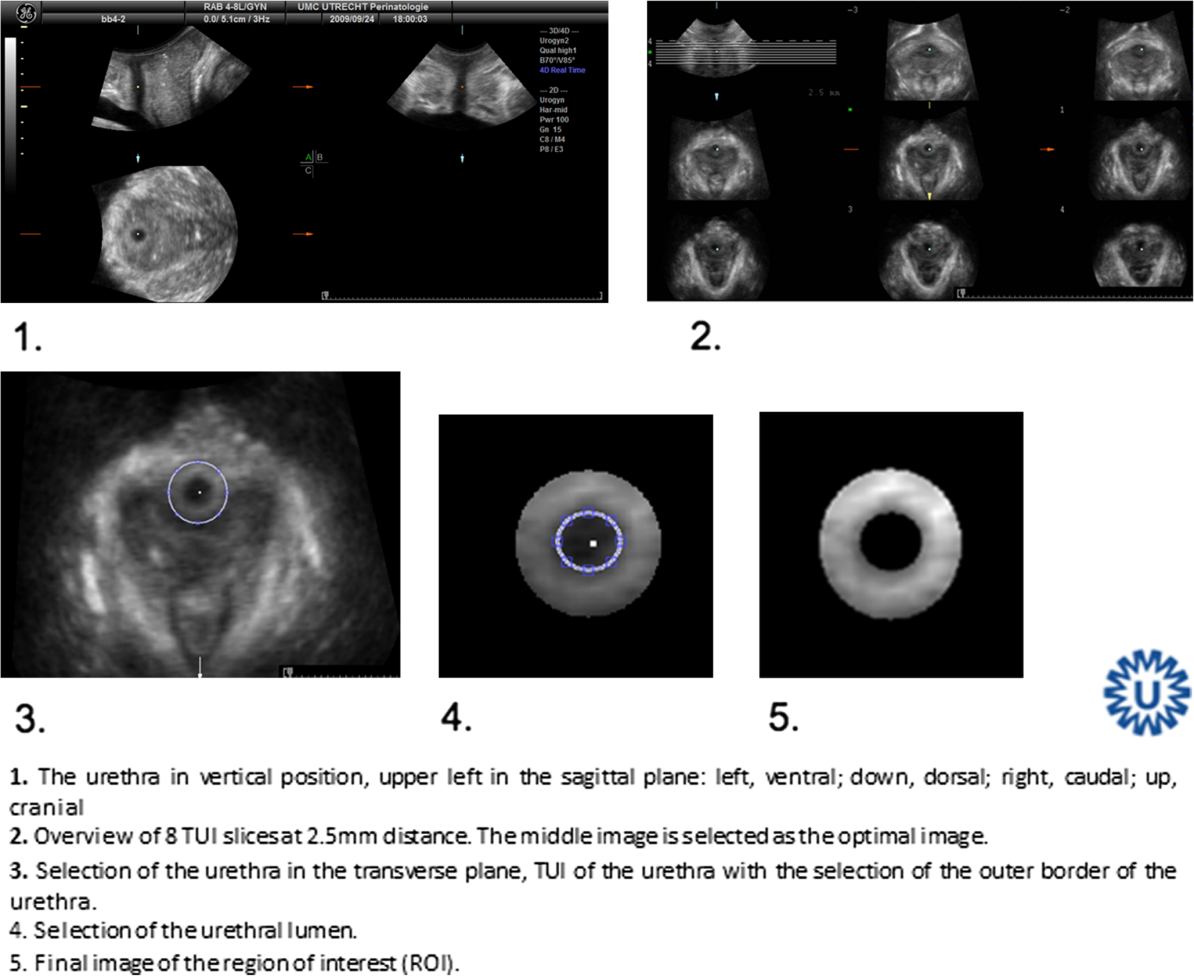
Delineation of the middle part of the urethra

A GE Voluson 730 Expert US system with a RAB 4-8-MHz curved array volume transducer (GE Healthcare, Hoevelaken, The Netherlands) was used, and settings that could influence echogenicity were set at constant values, as described by Scholten et al [17]. Settings were gain 15, power 100, harmonics mid, contrast 8, grey map 4, persistence 8, and enhance 3. Offline analysis was performed using 4D View 7.0 (GE Medical Systems Kretztechnik, Zipf, Austria) and Matlab® R2010a (MathWorks, Natick, MA, USA). Image analysis was performed on 4D views by determining a point at rest and at maximum pelvic floor muscle contraction. As shown in Fig. 1, in the sagittal plane, the urethra was positioned vertically by rotation around its axis (1), after which tomographic US images (TUI) with 2.5-mm slices were made in the transverse plane at approximately midurethral position (2). From these slices, the observer selected the image with optimal urethra visualization (2). With the use of Matlab® software (imellipse function), inner and outer borders of the midurethra were delineated (3, 4). In the remaining ring, consisting of urethral striated muscles, urethral smooth muscle, and submucosa containing vascular elements, the area of the urethral sphincter (cm^2^) and mean echogenicity [based on a greyscale image with a range from 0 (black) to 255 (white)] were calculated automatically (5).

Reliability testing, within and between observers, was performed on the 40 data sets at rest and on contraction. Intraobserver reliability was performed in two separate randomized orders to prevent recall bias. The second observer was instructed according to the protocol described and performed blinded measurements of all 40 data sets once. Measurements were obtained at separate times with no communication between the two observers.

Statistical analysis was performed using SPSS (21.0, 2012, Chicago, IL, USA). Intraclass correlation coefficients (ICCs) with 95% confidence intervals (95% CI) were calculated and interpreted using classification according to Landis and Koch (0.00–0.20 slight, 0.21–0.40 fair, 0.41–0.60 moderate, 0.61–0.80 substantial, 0.81–1.00 almost perfect concordance) [18]. Mean difference and limits of agreement (LOA) were determined according to the Bland–Altman method [19].

Changes in midurethral area and mean echogenicity over time were assessed with paired-samples *t* tests. A *p *value <0.05 was considered statistically significant.

## Results

Intraobserver reliability was substantial for measurements of midurethral area (ICC 0.77) and almost perfect for midurethral echogenicity (ICC 0.86) (Table 1). Interobserver reliability was moderate for midurethral area (ICC 0.53) and almost perfect for midurethral echogenicity (ICC 0.82) (Table 1).

**Table 1:** Intra- and interobserver reliability (40 data sets)

	Intraobserver reliability				Interobserver reliability			
	Mean	(SD)	ICC	(95% CI)	ICC	(95% CI)	MD	LOA
Area, cm^2^								
Rest	1.68	(0.52)	0.67	(0.23–0.70)	0.59	(0.22–0.79)	−0.01	−1.02 – 0.99
PFMC	1.57	(0.58)	0.84	(0.68–0.92)	0.46	(−0.03–0.72)	0.02	−0.61 – 1.26
Overall^a^	1.63	(0.55)	0.77	(0.63–0.85)	0.53	(0.26–0.70)	0.00	−1.12 – 1.13
Mean echogenicity								
Rest	100	(15)	0.78	(0.56–0.88)	0.81	(0.63–0.90)	−4	−25 – 17
PFMC	87	(18)	0.87	(0.75–0.93)	0.77	(0.54–0.88)	−6	−34 – 22
Overall^a^	93	(18)	0.86	(0.76–0.92)	0.82	(0.69–0.89)	−5	−29 - 20

*PFMC* pelvic floor muscle contraction,* ICC* intraclass correlation coefficient,* CI* confidence interval,* MD* mean difference,* LOA* limits of agreement

^a^At rest and on PFMC (*n* = 80)

**Fig. 2:**
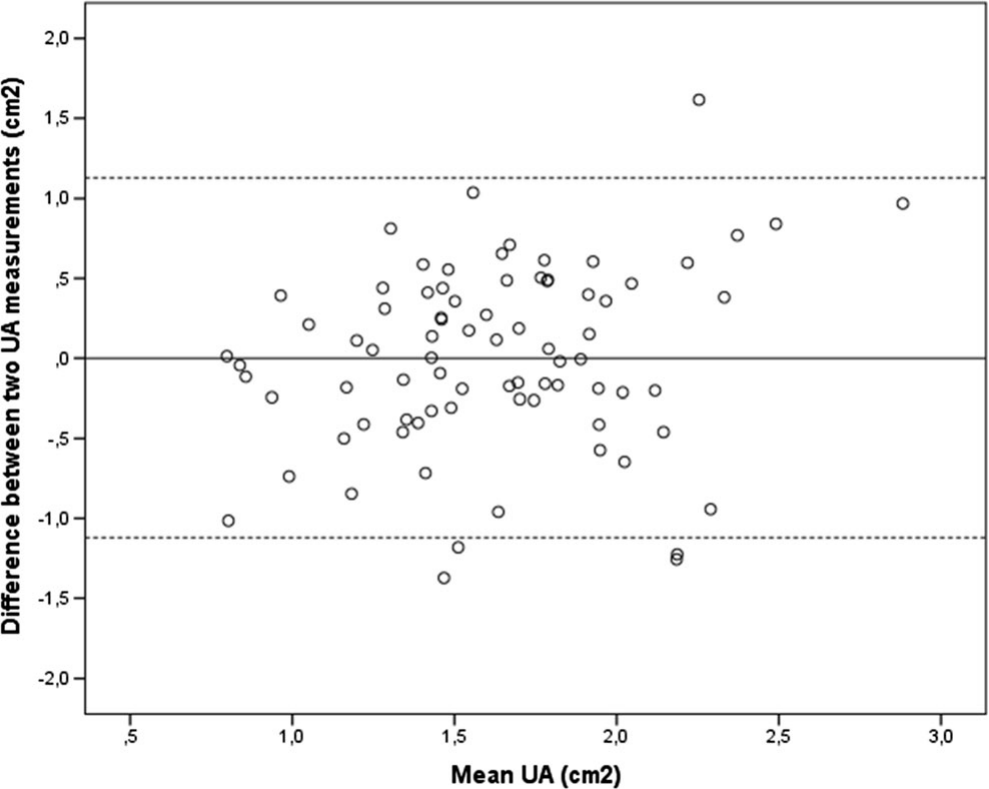
Bland–Altman plot of interobserver reliability of the urethral area (UA)

**Fig. 3:**
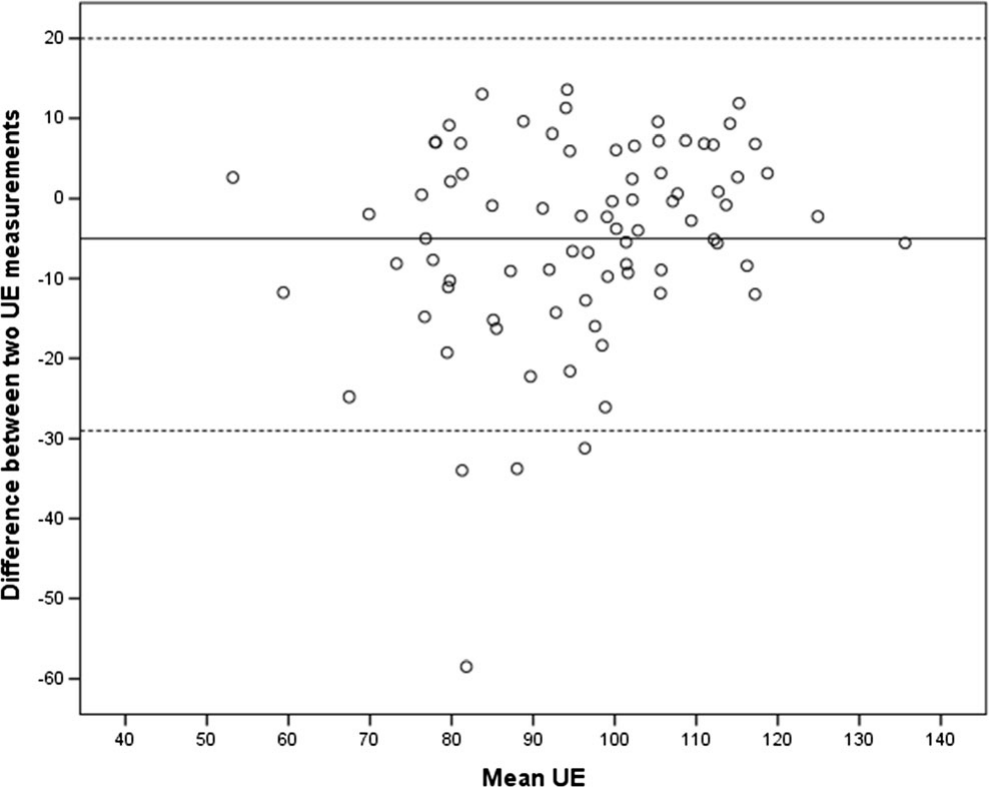
Bland–Altman plot of interobserver reliability of mean urethral echogenicity (UE)

Bland–Altman plots of interobserver reliability for midurethral area and echogenicity are shown in Figs. 2 and 3, respectively.

### Patients

Of the 280 women recruited initially, 26 were excluded. Reasons for exclusion were incorrect inclusion (*n =* 2) (one with a neurological disorder and one with a twin pregnancy), premature labor (19.9 weeks of gestation, *n =* 1), loss to follow up after 12 weeks of pregnancy (*n =* 17), and symphysis outside the view of the US image (*n =* 6). This left a data set of 254 women. The number of women presented in the figures reflects those who had complete and adequate US recordings for this particular item.

### Changes in midurethral area during pregnancy and after delivery

**Fig. 4:**
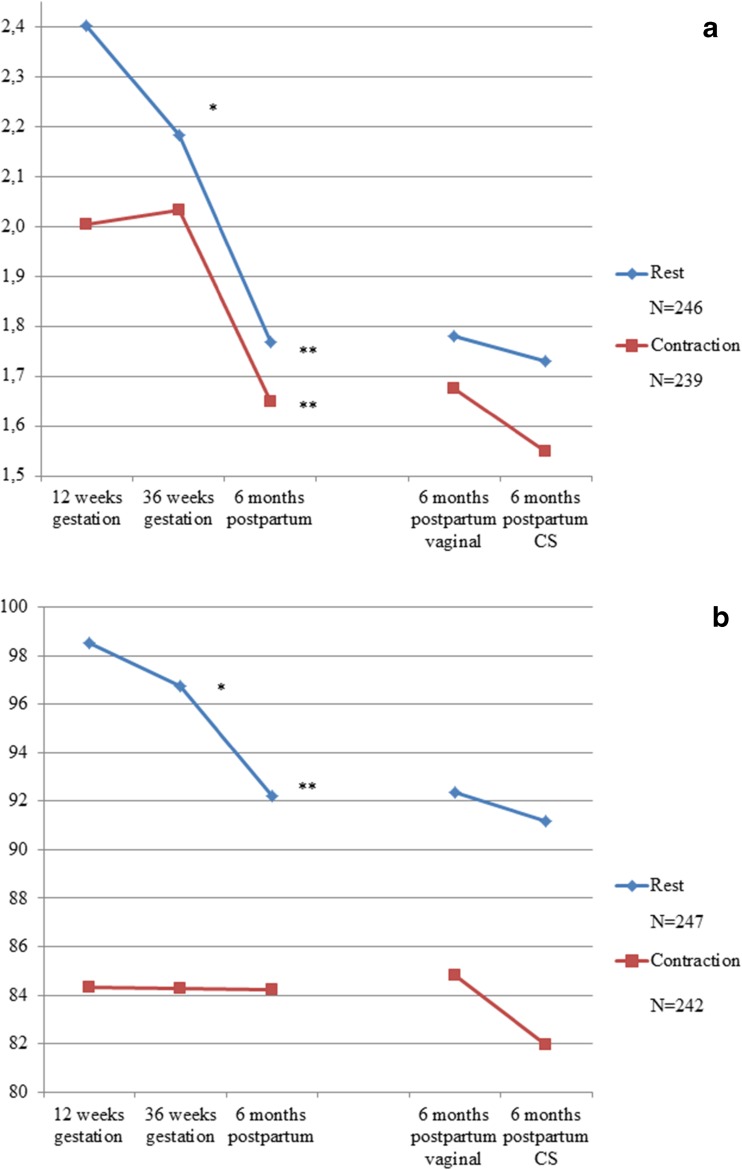
**a** Changes in midurethral mean area (cm^2^).** b** Changes in midurethral mean echogenicity

In Fig. 4a changes of the mean midurethral area at 12 and 36 weeks of gestation and 6 months after delivery are shown.

Mean midurethral area at rest was significantly smaller at 36 weeks than at 12 weeks of gestation (*p* < 0.05), but not on contraction (*p* = 0.21). At 6 months after delivery, midurethral area was statistically significantly smaller when compared with 12 and 36 weeks of gestation, both at rest (*p* < 0.05) and on contraction (*p* < 0.05). No statistically significant differences in mean midurethral area after delivery were found between vaginal delivery and caesarean section (at rest *p* = 0.47; on contraction *p* = 0.06).

### Changes in midurethral mean echogenicity during and after pregnancy

In Fig. 4b, changes in mean midurethral echogenicity at 12 and 36 weeks of gestation and 6 months after delivery are shown. Mean midurethral echogenicity changed significantly during pregnancy when the pelvic floor was at rest, but no difference was found when the pelvic floor was in contraction. A lower echogenicity at rest was seen at 6 months after delivery compared with at 12 and 36 weeks of gestation (*p* = 0.00 and *p* = 0.003, respectively). No significant differences in were found between vaginal delivery and caesarean section (at rest *p* = 0.62; on contraction *p* = 0.22).

## Discussion

This study shows that 3D/4D TPUS can be reliably used to assess midurethral area and mean echogenicity as a representation of the urethral sphincter. The protocol shows almost perfect interobserver reliability for midurethral echogenicity and moderate interobserver reliability for midurethral area. After delivery, mean area and echogenicity of the midurethra decreases significant compared with at 12 and 36 weeks of pregnancy, except for echogenicity during contraction. No significant differences in area and echogenicity were found between vaginal delivery and caesarean section.

A strength of this research is its prospective follow-up of 254 primigravid women at 12 and 36 weeks of gestational age and 6 months after delivery. In addition, the sample for the reliability study was chosen randomly, and intraobserver reliability was performed in two separate randomized orders to prevent recall bias. A limitation of our study is that we examined urethral area rather than volume [10, 12, 13, 16]. The strength of a muscle is mainly related to its volume and less to the area at a particular point. Therefore, from a functional point of view, it would be more appropriate to observe changes in volume instead of area. However, we measured area at its most functional point, e.g., midurethra, where closure pressure is greatest. Another possible limitation is that we included only nulliparous women with an intact pelvic floor, resulting in high-quality US images before delivery. However, we did not encounter any problems with visualizing the midurethra after delivery.

Digesu and coworkers calculated urethral sphincter volumes based on the sum of multiple axial cross-sectional areas [20]. They demonstrated good interobserver reliability of >0.6, which is comparable with our 0.59. It remains to be determined whether the time-consuming measurement of urethral volume would be clinically more relevant than single midurethral area measurements [20]. Various cadaver and imaging studies demonstrate that the urethral sphincter in women is located in the middle third of the urethra [10, 13, 16, 21–24]. In addition, maximal urethral closing pressure is observed in the middle section of the urethra [9]. Therefore, we feel confident that the position at which we measured area and echogenicity is clinical relevant.

We observed that during pregnancy, both urethral area and mean echogenicity in the resting state show significantly higher values compared with after delivery. The most plausible explanation is that pregnancy, with its increased levels of progesterone, causes an increase in intracellular and intramuscular fat storage [25]. As a consequence, the area will increase and the ratio between ECM components, including fat and muscle cells, will shift toward the ECM. As explained, the ECM will appear bright and muscle cells dark on US. An increase in ECM/muscle-cell ratio explains the higher echogenicity during pregnancy. This observation is consistent with the increase in puborectal muscle echogenicity we observed during and after pregnancy in the same cohort of women [26]. The decrease in urethral area during pelvic floor contraction can be attributed to compression of the urethra or active contraction of the urethral sphincter. Recently, Aljuraifani and coworkers demonstrated, by means of shear-wave elastography, that during pelvic floor muscle activation, the stiffness of the striated urethral sphincter increases [27]. This increase in stiffness represents muscle contraction, so it is plausible that voluntary pelvic floor muscle contraction also causes contraction of the urethral sphincter. During muscle contraction, the number of muscle cells/mm^3^ increases, which is consistent with the reduction in urethral echogenicity that we observed between rest and contraction. During maximum contraction, the decrease in echogenicity will be limited by the maximum number of muscle cells/mm^3^, which is consistent with our observation that echogenicity of the urethra on maximum contraction is the same at all three observation time points (12 and 36 weeks of gestation and 6 months after delivery).

Although there was a statistically significant decrease in urethral area and echogenicity after delivery, we could not demonstrate a difference between women who had a vaginal delivery versus cesarean section. One explanation is that vaginal delivery itself does not have the same damaging effect on the urethral sphincter as it has on the pelvic floor musculature. An alternative explanation is that pregnancy produces permanent changes in urethral sphincter composition, regardless the mode of delivery. In our previous study of the levator hiatus dimensions during and after delivery, we demonstrated a persistent increase in distensibility of the levator hiatus at 6 months after delivery for both vaginal and cesarean deliveries [3].

The clinical merit of our method of measuring urethral area and echogenicity needs to be further studied. For instance, the association with complaints of urinary incontinence and the response to treatment is an interesting area.

In conclusion, this study presents a reliable method to assess transverse midurethral area and mean echogenicity using TPUS. With this protocol, we show that area and echogenicity, except for during contraction, of the midurethra significantly decreases after pregnancy.

## Appendix

**Table 2:** Midurethral area (UA) values during pregnancy and after delivery

	12 weeks’ gestation	36 weeks’ gestation	6 months postpartum		
No.	*mean (SD)*	*mean (SD)*	*Total group*	Vaginal delivery	CS
			*mean (SD)*	*mean (SD)*	*mean (SD)*
**UA** (in cm^2^)					
Rest (246)	2.40 (0.66)	2.18 (0.61)*	1.77 (0.40)**	1.78 (0.41)	1.73 (0.37)
Contraction (239)	2.00 (0.61)	2.03 (0.62)	1.65 (0.39)**	1.68 (0.39)	1.55 (0.39)

*SD* standard deviation, *CS* cesarean section

**P* ≤ 0.05 vs 12 weeks’ gestation

***P* ≤ 0.05 vs 12 and 36 weeks’ gestation

**Table 3:** Midurethral mean echogenicity (UE) values during pregnancy and after delivery

	12 weeks’ gestation	36 weeks’ gestation	6 months’ postpartum		
No.	*mean (SD)*	*mean (SD)*	*Total group*	Vaginal delivery	CS
			*mean (SD)*	*mean (SD)*	*mean (SD)*
**UE**					
Rest (247)	98.51 (12.77)	96.72 (13.58)*	92.20 (13.87)**	92.35 (13.65)	91.19 (14.81)
Contraction (242)	84.31 (15.00)	84.26 (14.63)	84.20 (13.13)	84.81 (12.79)	81.97 (14.26)

*SD* standard deviation, *CS* cesarean section

**P* ≤ 0.05 vs 12 weeks’ gestation

***P* ≤ 0.05 vs 12 and 36 weeks’ gestation
